# Tackling Biofilm-Forming Pathogens: A Challenge to Overcome in the Fight Against Infectious Diseases

**DOI:** 10.3390/pathogens15050493

**Published:** 2026-05-03

**Authors:** Elenoire Sole, Giuseppe Motta, Federica Marcoli, Angelina Midiri, Cinzia Sindona, Liliana Imbesi, Giuseppe Mancuso, Mohamed Zemzem, Carmelo Biondo

**Affiliations:** Department of Human Pathology, University of Messina, 98125 Messina, Italy; elenoiresole@icloud.com (E.S.); giuseppe.motta@studenti.unime.it (G.M.); federica.marcoli@studenti.unime.it (F.M.); amidiri@unime.it (A.M.); cinzia.sindona@studenti.unime.it (C.S.); liliana.imbesi@studenti.unime.it (L.I.); mancusog@unime.it (G.M.); mohamed.zemzem@studenti.unime.it (M.Z.)

**Keywords:** hospital-acquired infections, AMR, MDR, biofilm resilience

## Abstract

Microorganisms can aggregate and organise into structured communities embedded within an exopolysaccharide-based matrsix, which serves as a protective barrier and a functional environment around microbial cells. The formation of biofilms is widely recognised as a pivotal factor in bacterial virulence, impeding the efficacy of antimicrobial agents and hindering immune responses, whilst concomitantly contributing to the development of antimicrobial resistance and the onset of persistent infections. Biofilm formation is a tightly regulated and dynamic process, controlled by quorum-sensing mechanisms and profoundly influenced by environmental factors and nutrient availability. The objective of this review is to elucidate the significance of biofilms in clinical settings, with a particular focus on their role in the pathogenesis of infectious diseases. Particular attention is devoted to biofilm-associated infections and infections related to invasive medical devices, with a particular emphasis on the most prevalent microbial pathogens, which include *S. aureus, S. epidermidis, P. aeruginosa, E. coli, K. pneumoniae, A. baumannii* and various species of *Candida*. Furthermore, the present review encompasses biofilm-associated chronic infections, conditions manifesting in predisposed patients, including individuals affected by cystic fibrosis. This review further examines the most recent strategies for combating antibiotic resistance in bacterial biofilms. This review focuses on recent biofilm pathogenesis advancements, with a focus on diagnosis challenges and the need for new ways to disrupt biofilm integrity.

## 1. Introduction

Bacteria are conventionally studied as single-celled, planktonic organisms. However, it has been demonstrated that microorganisms have the capacity to aggregate and organise themselves into structured communities embedded within an exopolysaccharide-based matrix. This matrix serves a dual function, acting as a protective barrier and a functional environment around microbial cells. In their natural habitat, the majority of these organisms reside in organised, multicellular communities known as biofilms. Bacteria alternate between planktonic and sessile states, enabling them to adapt quickly to changing environments [1,2]. Many microorganisms form microbial consortia within an exopolysaccharide (EPS) matrix on both biotic and abiotic surfaces providing protection against unfavourable conditions. Although almost all bacteria are capable of forming biofilms, the major causative agents of biofilms on various medical devices are *Staphylococcus aureus, MRSA, Staphylococcus epidermidis, Escherichia faecalis, Klebsiella pneumoniae, Pseudomonas aeruginosa, Proteus mirabilis, Acinetobacter baumannii, Enterobacter cloacae, Mycobacterium* spp., *Corynebacterium* spp., *Moraxella catarrhalis, Burkholderia cepacia complex* and *C. albicans* [3,4,5]. The notion of biofilms can be traced back to the late 17th century, when Antonie van Leeuwenhoek, the inventor of the microscope, first documented the presence of microorganisms in the dental plaque extracted from his teeth [6]. However, it was not until 1978 that the term "biofilm" was coined by Costerton and his colleagues [7]. Biofilms are defined as complex structures composed of an aggregate of microbes that are encased in a self-produced and protective EPS. The latter is composed of a variety of polymers, including polysaccharides, proteins, and extracellular DNA. These polymers are involved in the adhesion of cells to one another and to a surface, a process that is often critical for various biological functions, including high tolerance to antibiotics, biocides, desiccation and the host immune system [3,5]. Biofilm communities are frequently polymicrobial in nature, comprising a mixture of Gram-positive and Gram-negative bacteria, including, but not limited to, *Staphylococcus aureus* and *Pseudomonas aeruginosa* [8]. In certain circumstances, these communities can be comprised of a combination of bacterial and fungal associations, such as *S. aureus* and *Candida albicans*, which serves to further complicate the therapeutic management of these communities [9]. In such contexts, it is imperative that antimicrobial therapy is effective against all pathogenic species involved, thereby posing an additional challenge for the treatment of patients [10]. Despite the long-recognised importance of biofilms in natural environments, as evidenced by their prevalence in diverse ecological niches such as seawater, groundwater and soil, research has historically focused on bacteria that are found living freely in the environment [3,11]. It has been established that biofilms enhance microbial survival and functional adaptability, thereby allowing bacteria to interact and communicate in ways that are not possible for free-living bacteria [12]. Moreover, it has been demonstrated that biofilms enable the transfer of genetic information and horizontal gene transfer [13]. This process has been shown to promote the survival of antibiotic-tolerant cells, which then repopulate the biofilm following the removal of antibiotics [14]. Several studies have shown that biofilm is refractory to antibiotic action due to multiple tolerance mechanisms collectively referred to as ˝phenotypic resistance˝, including reduced antibiotic penetration, low oxygen tension within the biofilm core, and the differential expression of resistance-related genes [15,16]. Furthermore, biofilm-related diseases are a type of slow-developing infection that frequently persists despite immune system activation [17,18]. According to reports from the National Institutes of Health (NIH), approximately 65% of microbial infections and up to 80% of chronic infections are associated with biofilm formation, underscoring the urgent need to develop effective antibiofilm strategies [15,19]. The present review aims to provide a comprehensive examination of biofilm-associated infections within healthcare environments. The focus is on the following key areas: medical device infections, antibiotic resistance, prevalence, treatment and prevention. A literature search was conducted using primary biomedical databases to support the objective. The search strategy combined keywords related to biofilm- and device-related infections, antibiotic resistance, quorum sensing, multidrug-resistant (MDR) pathogens and bacteriophages.

## 2. The Biofilm Life Cycle

**Figure 1:**
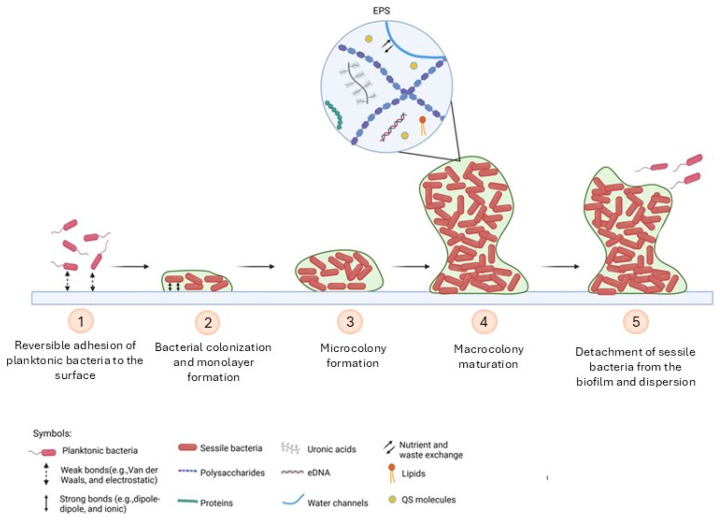
The five stages of biofilm formation. The formation of biofilms is a cyclic process initiated following surface contact by single planktonic cells. In the initial phase of adhesion, planktonic bacteria reversibly attach to a surface through electrostatic forces and Van der Waals interactions. In the subsequent phase, bacterial colonisation of the surface is observed, resulting in the formation of a monolayer. The formation of this layer is facilitated by dipole–dipole and ionic interactions, which ensure a robust and stable adhesion. The third phase is characterised by the formation of microcolonies, which subsequently mature into macrocolonies in the fourth phase. In the final phase, bacteria detach from the biofilm and disperse. This process initiates a new cycle or is prompted by stress factors, including changes in temperature, pH levels, and depletion of nutrients or oxygen.

Biofilms are conventionally defined as organised aggregates of microorganisms (ranging from 10^8^ to 10^11^ cells per gram) that are held together by a self-produced extracellular matrix [3]. From a structural standpoint, biofilms can be considered a highly hydrated and complex system, primarily composed of microbial cells (approximately 2–5%), water (up to 97%), polysaccharides (1–2%, including cellulose and sucrose-derived fructans and glucans), proteins (<1–2%, including enzymes), extracellular DNA (eDNA <1–2%), and membrane vesicles [19,20,21]. The collective nature of these elements contributes to the biofilm’s mechanical stability and intercellular communication [10]. Many in vitro systems have been used to examine biofilm formation. These systems typically use shaken, well-mixed cultures with a single seeding event of planktonic cultures, where a measured number of cells is introduced [21]. Moreover, the transformation of individual cells into sessile biofilm communities has been the focus of extensive research in closed in vitro systems, wherein no influx of new cells has been introduced during the process of biofilm formation [21,22]. The extant “5-step biofilm model” has been formulated on the basis of the aforementioned studies [23]. The formation of biofilms begins when bacteria transition to a sessile lifestyle, sticking to a surface. This initial adhesion stage is followed by an early development phase, during which reversible and irreversible attachment is observed (attachment is unstable, and cells often return to the original state). Reversible adhesion has been observed to occur on both abiotic and biotic surfaces [24]. The phenomenon is influenced by a number of environmental and physicochemical parameters, including pH, temperature, nutrient availability, gravitational forces and Brownian motion [25,26]. The adhesion of bacterial cells to non-living surfaces represents the initial stage in the formation of biofilms. This phenomenon is primarily driven by weak electrostatic and hydrophobic interactions [27]. It is known that bacterial cells possess a net negative surface charge, attributable to the presence of amino, carboxyl and phosphate groups that facilitate interaction with positively charged surfaces. In Gram-negative bacteria, this surface charge is largely determined by the structure of lipopolysaccharide, whereas in Gram-positive organisms, teichoic acids play a pivotal role [3,28]. Conversely, the process of adhesion to biotic surfaces is dependent upon the expression of adhesins that are capable of binding host components, such as collagen [29]. Moreover, the significance of fimbriae and flagella in the initial attachment phase has been extensively established [30]. A maturation phase then ensues, characterised by irreversible attachment [31]. During this stage, the bacteria secrete a viscous EPS, which functions as an adhesive agent, thereby irreversibly anchoring the cells to the substrate. Irreversible adhesion is stabilised by a combination of hydrogen bonds, ionic and covalent interactions, and dipole–dipole forces [24,31]. Intracellular second messengers, particularly bis (3′–5′)-cyclic dimeric guanosine monophosphate (c-di-GMP), play a central regulatory role by reducing flagellar motility and promoting EPS synthesis [32,33]. Furthermore, evidence indicates that the levels of c-di-GMP within cells exhibit significant fluctuations during the processes of attachment and detachment. This dynamic equilibrium is believed to play a crucial role in coordinating the transition between the planktonic and sessile states [16,34]. This phase has been the focus of extensive research and detailed description in a model based on *P. aeruginosa* biofilm formation [33]. In this phase, there is activation of numerous genes that are implicated in various processes, including the biosynthesis of lipopolysaccharide core and the biosynthesis of the exopolysaccharide alginate [35]. In addition, a range of other processes have been identified, including Psl matrix biosynthesis and the activation of genes linked to antibiotic resistance, such as *β-lactamase, SagS* and *BrlR* [21,36]. The development and maturation of biofilms are also subject to strict regulation by QS, a cell-density-dependent communication system based on the production and detection of autoinducers [34,37]. This system coordinates gene expression at the population level and modulates the production of exopolysaccharides (EPSs), the release of extracellular DNA, and dispersal mechanisms, that is, the shift between the biofilm-associated state and the planktonic state is facilitated, thereby promoting dispersal when resources become limited or population density is high [38]. Subsequent to the attachment process, the development of a three-dimensional structure is observed, characterised by the presence of heterogeneous microcolonies interspersed with water channels. This dynamic process effectively establishes a route through which nutrients and waste by-products can be delivered [39]. This, in turn, enables the ongoing colonisation and maturation of the embedded bacteria [40]. Mature biofilms may acquire mushroom- or tower-like architectures. Microorganisms are organised within these structures according to metabolic activity and oxygen availability, often forming multilayered structures consisting of a basal microbial layer, an intermediate regulatory zone, and an outer layer enriched with cells poised for release [41]. As biofilms evolve their three-dimensional structure, bacteria residing at different locations within the biofilm are subjected to variable concentration gradients of nutrient resources, oxygen, and extracellular signalling molecules [40]. This is evidenced by the expression of genes associated with oxygen deprivation, general stress, stationary phase conditions, as well as nutrient stress and slow growth by resident bacteria near the base compared to other subpopulations within the biofilm [42]. The EPS matrix not only stabilises this architecture but also confers protection against antibiotics, oxidative stress, metal cations, and the innate immune response. For instance, the process of eliminating bacteria by neutrophils is dependent on the disruption of the biofilm structure, which can extend to dimensions far exceeding the phagocytic capacity of these cells [38,43]. Furthermore, biofilm-associated proteins, frequently characterised by conserved amyloid-like repeats, contribute to processes such as adhesion, immune evasion, and nutrient transport through structured channels, representing promising potential therapeutic targets [41]. In the final stage, known as the dispersion phase, a percentage of bacteria detach from the biofilm, thus enabling it to spread and colonise new environments [44]. Finally, bacteria, whether isolated or in the form of microaggregates, possess the capacity to leave the biofilm structure, return to the planktonic mode of growth (by a process referred to as dispersion), which leads to bacterial dissemination and the colonisation of new environments, thereby initiating a new life cycle [31] (Figure 1). Dispersion may be triggered by a number of factors, including, but not limited to, nutrient depletion, overpopulation, or increased competition. This process represents a critical step in the persistence and spread of biofilm-associated infections [3].

## 3. Biofilms in Healthcare-Associated Infections

**Figure 2:**
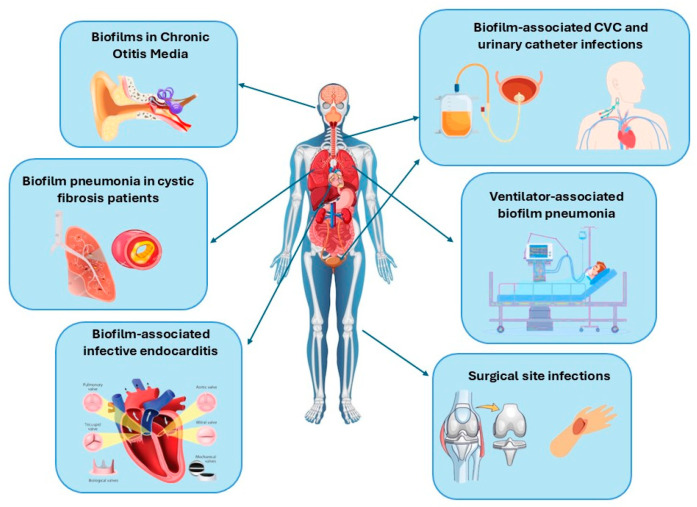
Biofilm-related infections.

In clinical settings, the process of biofilm formation is of central importance in the pathogenesis of a variety of healthcare-associated infections (HAIs), including central line-associated bloodstream infections (CLABSIs), catheter-associated urinary tract infections (CAUTIs), ventilator-associated pneumonia (VAP), surgical site infections (SSIs), and gastrointestinal infections caused by *Clostridioides (Clostridium) difficile* [45] (Figure 2).

The following schematic representation illustrates the spectrum of infections attributable to biofilm:

- Chronic otitis media (commonly involving *Pseudomonas aeruginosa* and *Staphylococcus aureus*).
- Pneumonia in patients with cystic fibrosis (*P. aeruginosa, Burkholderia cepacia complex*).
- Infective endocarditis (*S. aureus*, *Streptococcus* spp.).
- Biofilm-based central line-associated bloodstream infections (*S. epidermidis, S. aureus, E. faecalis, P. aeruginosa).*
- Urinary catheter-associated infections (*S. aureus, Enterococcus* spp., *Candida* spp.).
- Ventilator-associated pneumonia (*P. aeruginosa, Acinetobacter baumannii*).
- Surgical site infections (*S. aureus, Escherichia coli*).
- Impact of biofilm formation on recurrent *C. difficile* infections.

These infections are frequently characterised by persistence and resistance, which can be attributed to biofilm-mediated antimicrobial tolerance.

The National Institutes of Health (NIH) has established a correlation between the formation of biofilms and a range of health complications. Specifically, the prevalence of biofilms has been linked to 65% and 80% of microbial infections and chronic illnesses, respectively [15]. The number of infections associated with the formation of biofilms continues to rise, a phenomenon that can be attributed in part to the demonstration that pathogenic biofilm aggregates can form without adhering to a surface [5]. Conventional research approaches have historically concentrated on the growth of biofilms on solid surfaces. However, it is now widely acknowledged that pathogens can also form biofilm-like aggregates within the liquid volume of biological fluids. Such non-device-related microbial biofilm infections encompass a range of conditions, including otitis media, cystic fibrosis, infective endocarditis and chronic inflammatory disorder [15,46]. The following section provides an overview of device-associated and non-device-related biofilm infections.

### 3.1. Key Aspects of Biofilms in Chronic Otitis Media

Chronic otitis media (COM) is defined as an inflammatory process affecting the middle ear, usually manifesting in the form of ear discharge (otorrhoea) and hearing loss (hypoacusis). The symptoms have been demonstrated to have a considerable effect on patients’ quality of life, particularly in children [47]. As demonstrated in previous studies, the process of biofilm formation is a pivotal factor in the persistent nature of the disease [48]. Furthermore, it has been observed that this phenomenon is a contributing factor to the development of antimicrobial resistance, thus resulting in treatment failure [49]. Biofilms have been detected in up to 69.9% of cases of COM, with *Pseudomonas aeruginosa* (40%), *Staphylococcus aureus* (30%), *Haemophilus influenzae* and *Moraxella catarrhalis,* being the principal agents responsible for the formation of biofilms on middle ear mucosa [50,51]. In contrast to planktonic cells, biofilms function as a protected microecosystem that protects bacteria from the hostile environment. This phenomenon has been shown to result in elevated levels of antibiotic tolerance, thereby enabling bacteria to evade immune defences and inducing a state of chronic inflammation [52]. Moreover, the role of biofilms has been extensively documented in the pathogenesis of adhesive otitis media. This condition is characterised by the accumulation of viscous fluid within the middle ear, creating a conducive environment for bacterial proliferation on the mucosa, despite the absence of positive bacterial cultures and the administration of antibiotics [48]. The presence of bacterial aggregates in the middle ear cavity, as evidenced by confocal laser scanning microscopy, further substantiates the hypothesis that biofilms have the capacity to form in the middle ear fluid, in addition to their capacity to develop on the mucosa of the middle ear [53]. A dangerous form of chronic otitis media that frequently results from unresolved acute or chronic Eustachian tube dysfunction is known as chronic suppurative otitis media (CSOM) [54]. This infection has been identified as a prevalent condition among children worldwide, representing one of the most common chronic infectious diseases in this age group [47,55]. The consequences of this condition are the primary cause of preventable hearing loss, a problem that is especially prevalent in lower-income countries [56]. Moreover, the condition is characterised by chronic inflammation of the middle ear and mastoid cavity, which is typically accompanied by persistent (often weeks-long) otorrhea through a non-intact tympanic membrane [57]. In cases of extreme severity, this ear disease has the potential to result in significant complications, including those affecting the intracranial region [47]. Its association with *Pseudomonas aeruginosa* and *Staphylococcus aureus* infections is well documented, and it has been shown to result in a range of severe pathologies, including but not limited to brain abscess, meningitis and lateral sinus thrombophlebitis [58,59]. The role of biofilms in the pathogenesis, persistence and antibiotic resistance of CSOM has been confirmed by numerous research studies. In particular, studies utilising scanning electron microscopy have identified a high percentage of middle ear tissue samples from CSOM patients as being positive for biofilm-producing bacteria [60,61]. Furthermore, it is hypothesised that biofilms could be the cause of the relapsing nature of CSOM, despite the administration of appropriate antibiotic therapy. The concentration of antibiotics necessary for the eradication of biofilms is not achieved by orally administered antibiotics in the middle ear [61]. Estimates indicate that CSOM has a global prevalence exceeding 20 million cases. Notably in industrialised nations, approximately 80% of CSOM cases emerge in preschool children, specifically those below the age of three, with an estimated 40% of these individuals experiencing relapses [47]. The primary risk factors that have been identified are as follows: an immature immune system, previously untreated acute otitis media, poor hygiene, limited access to hospitals, inadequate health resources, and malnutrition [47,62].

### 3.2. Biofilm in the Cystic Fibrosis Lung

Biofilms represent a significant concern for patients suffering from cystic fibrosis (CF), the most common inherited lethal genetic disorder affecting Caucasian populations, with approximately 35,000 children and adults affected in Europe [63]. CF is a hereditary condition arising from mutations in the cystic fibrosis transmembrane regulator (CFTR) gene. Mutations have been demonstrated to induce defective chloride transport and secretion, resulting in mucus release and obstruction of mucociliary clearance within the respiratory tract. This, in turn, has been shown to cause persistent bacterial colonisation, chronic inflammation, and chronic lung damage [64]. In the absence of treatment, this may result in chronic polymicrobic infections, most commonly in the respiratory system, with lung infection being the primary cause of morbidity and mortality [65]. *Pseudomonas aeruginosa* biofilms have been demonstrated to manifest elevated antibiotic tolerance and enhanced resistance to host responses, thus ascribing this microorganism the primary causative agent of CF lung infections, despite the prevalence of *Staphylococcus aureus* and *Haemophilus influenzae* as primary colonisers of the airways [66]. *S. aureus* and *H. influenzae* also form biofilms, which help them evade the body’s defences. This makes them the most prevalent opportunistic pathogens identified in the lungs of children diagnosed with cystic fibrosis [67]. *P. aeruginosa* possesses the capacity to infect tissues and evade the immune defences of the host organism via a plethora of virulence factors, encompassing exotoxins (e.g., ExoU, ExoS), pigments (e.g., pyoverdine, pyochelin), and motile structures (e.g., flagella, pili) [68]. Furthermore, this pathogen has the potential to undergo both genotypical and phenotypical mutation, including the conversion to its mucoid form. The latter is a hallmark of biofilms, wherein the EPS matrix functions as a protective barrier for the cells within the biofilm, thereby markedly augmenting their resistance to external agents, including antibiotics [69]. While *H. influenzae* and, at a slightly subsequent phase, *S. aureus* are identified as the predominant etiological agents during the childhood period, *Pseudomonas aeruginosa* is recognised as the principal etiological agent of bronchopulmonary infections in adolescence and adulthood [70]. However, this finding does not preclude the possibility that it has been isolated in some cases in children during their initial few months of life [71]. *P. aeruginosa* exhibits a pattern of sporadic or intermittent colonisation, whereby positive and negative cultures alternate, leading to the development of chronic colonisation and recurrent infection. In these stages, predominantly mucoid strains of *P. aeruginosa* are isolated [58]. Other pathogens which have been isolated from the lungs of patients diagnosed with CF include: *Achromobacter xylosoxidans, Streptococcus milleri, Ralstonia* spp., *Pandorea* spp., *Stenotrophomonas maltophilia* and *Mycobacterium* spp. (particularly *M. abscessus* [72]. These bacteria, in conjunction with oral commensals that become opportunistic pathogens—specifically *Rothia mucilaginosa* and *Gemella haemolysans*—are capable of forming biofilms that protect them from the action of antibiotics and host defences [63].

### 3.3. Biofilm in Infective Endocarditis

Infective endocarditis (IE) is a severe condition caused by bacteria that have colonised the inner lining of the heart, as well as the valves that separate the four cardiac chambers [73]. This infection represents the fifth most common worldwide in terms of incidence, and despite continuous advances in therapeutic, diagnostic and patient management strategies, its incidence continues to increase [74]. The most prevalent symptom of infective endocarditis is fever, which is observed in approximately 90% of patients, frequently accompanied by chills, arthralgia, myalgia, weight loss, splenomegaly and petechiae [75]. The primary etiological agent implicated in IE is *Staphylococcus aureus*, particularly methicillin-resistant strains (MRSA), which is characterised by its capacity to produce biofilm [76]. The latter is a pivotal aspect of MRSA’s capacity to invade, disseminate and evade antimicrobial treatments. As demonstrated in earlier studies, the extracellular polymeric substance (EPS) matrix exerts an effect that provides protection not only against antibiotic treatment, but also against host immune defences [76]. Furthermore, the presence of certain risk factors has been demonstrated to predispose patients to developing complications. These include prior cardiac surgery and the presence of intracardiac prosthetic material, such as prosthetic valves, cardiac implants, and central venous catheters [76]. The process of *Staphylococcus aureus* biofilm formation is a complex phenomenon that is subject to strict regulations by a complex genetic regulatory network. This network orchestrates the transition from free-living (planktonic) cells to a protected, sessile community, principally through the coordination of three primary systems: the ica locus (polysaccharide production), the agr system (quorum sensing/dispersal), and the cid/lrg regulatory networks (extracellular DNA release) [77]. Notwithstanding the advances in medical and surgical treatment, the infection continues to carry a high morbidity and mortality rate. In-hospital mortality rates range from 15% to 20%, and the 1-year mortality rate approaches 40% [78]. In the context of this infection specifically, there is a potential for fatal complications to occur, the consequences of which may range from cardiac arrest and valvular damage to systemic embolisation and neurological deficits [76]. The diagnosis is established through a combination of microbiological analyses, imaging techniques, and clinical findings, in accordance with the Modified Duke Criteria [79]. Early diagnosis is imperative, as it has been demonstrated to have a substantial impact on the ultimate course of the infection. The management of patients with this condition necessitates prolonged antibiotic therapy and, when deemed necessary, surgical intervention. First-line antibiotics for the treatment of IE include vancomycin and daptomycin [80].

### 3.4. Biofilm-Based Central Line-Associated Bloodstream Infections

Although central venous catheters (CVCs) are an essential medical device, they have been associated with a significant incidence of infection, accounting for 87% of cases of bloodstream infections [81]. This phenomenon is particularly evident among patients in critical condition, those undergoing oncological treatment, and those requiring parenteral nutrition or haemodialysis. Estimates suggest that 250,000 to 400,000 cases of central line-associated bloodstream infections (CLABSIs) occur annually in the United States. The estimated annual treatment cost of these infections ranges from 296 million to 2.3 billion US dollars, and the mortality rate is between 12 and 25 per cent [82,83]. A wide range of patient-related risk factors have been identified, including the presence of comorbidities, diabetes mellitus, immunocompromised status, chronic kidney disease and other underlying conditions [84]. The formation of biofilms on catheters is a process that typically occurs with a host-derived conditioning layer deposited onto the surface. This layer is constituted by plasma proteins, including fibrinogen and fibronectin, which function as a structural framework, thereby enabling the initial, reversible microbial adhesion [85]. This initial interaction subsequently becomes irreversible through the action of bacterial cell wall–associated proteins that mediate stable attachment. *Staphylococcus aureus* has the capacity to produce a wide range of surface-anchored adhesins, which facilitate the bacteria’s adhesion to host extracellular matrix components, including fibrinogen, fibronectin, vitronectin, and collagen [86]. Furthermore, the bacteria are capable of producing proteins that have been demonstrated to facilitate the development of biofilms, such as Bap, SasG, Aap, EmbP, FnBP-A, and FnBP-B [87]. In the initial phase of the pathogenesis of *E. coli*, the bacterium adheres to the surface of the catheter via type I pili. This is followed by the formation of a biofilm, which is a key feature of the ability of the bacterium to cause disease [45]. In contrast, *P. aeruginosa* employs type IV pili to facilitate movement across surfaces and to establish microcolonies, which are crucial for the maturation of the biofilm. The latter functions as a persistent reservoir of pathogens, with the potential to result in intermittent bacteremia through the detachment of planktonic cells into the bloodstream [88]. Moreover, it has been demonstrated that this microenvironment is capable of conferring antibiotic resistance and protecting against host immune defences [68]. The most prevalent microorganisms associated with CLABSI comprise *Staphylococcus epidermidis, Staphylococcus aureus, Enterococcus faecalis, Pseudomonas aeruginosa, Candida albicans, Propionibacterium, Anaerococcus, Peptoniphilus* and *Bacteroides* [89,90]. All of these microorganisms possess the capacity to generate the EPS matrix. The infections caused by these microorganisms are primarily acquired during the insertion, handling and management of catheters. Failure to adhere to aseptic technique, or the repeated manipulation of catheter hubs, has been demonstrated to facilitate contamination of the insertion site or intraluminal surface by commensal or environmental bacteria [91].

### 3.5. Biofilm-Based Catheter-Associated Urinary Tract Infections

Urinary tract infections (UTIs) are the most prevalent type of infection globally, with an estimated 150 million cases reported annually [92]. Two broad categories emerge: community-acquired infections, which occur sporadically in the general population, and hospital-acquired infections. Community-acquired UTIs typically affect otherwise healthy and immunocompetent individuals, whereas nosocomial infections occur predominantly in hospitalised patients, who are often immunocompromised, bedridden, or affected by urinary tract obstruction or retention, or who are exposed to indwelling urinary catheters [92]. Patient-related factors, including sex, age, and the presence of comorbidities, have been identified as factors that may influence susceptibility [93]. Catheter-associated urinary tract infections (CAUTIs) are the most common healthcare-associated infections, with an estimated incidence of approximately 1 million cases per year in the United States alone [94]. A CAUTI is defined as a UTI occurring in a patient with an indwelling urinary catheter, either while the catheter is in situ or within 48 h of its removal, with a urine culture yielding ≥10^5^ colony-forming units (CFU)/mL of a bacterial species. The most frequently identified pathogens include *Escherichia coli*, *Enterococcus* spp., *Klebsiella pneumoniae, Candida* spp., *Staphylococcus aureus, Proteus mirabilis, Pseudomonas aeruginosa*, and Group B *Streptococcus* [92,95]. The expression of multiple virulence factors by these microorganisms enables adhesion and the formation of biofilms, with a direct proportionality to the duration of catheterisation. Bacterial colonisation of the catheter may result from ascending migration of organisms from the perianal area, improper management of the catheter by health care professionals, or prolonged exposure to the device [96]. It has been demonstrated that the duration of catheterisation is associated with an increased risk of bacteriuria, with a range of 3–7% increase in risk observed per day [94]. Consequently, whenever feasible, catheter use should be avoided, and if used, must be limited to clear clinical indications and maintained for the shortest feasible duration. Replacement is typically recommended every four weeks and strict attention to hygiene is absolutely essential [97]. CAUTIs are a particular concern as they have the potential to result in secondary bloodstream infections and promote antibiotic resistance due to the formation of biofilms. The principal clinical manifestations encompass a range of symptoms, including fever, cystitis, urethritis, acute pyelonephritis, renal scarring, stone formation, and bacteremia [45,94,98].

### 3.6. Biofilm in Ventilator-Associated Pneumonia

Ventilator-associated pneumonia (VAP) is a complication that develops at least 48 h after exposure to an invasive mechanical ventilation device [99]. The reported incidence of VAP ranges from 5% to 40%, depending on the clinical setting and diagnostic criteria, while mortality is estimated to be around 10%, particularly in intensive care units. ICU mechanical ventilation is an invasive technique delivered through an artificial airway that is either an endotracheal tube for short-term use or a tracheostomy tube for long-term support [100]. In both cases, this is regarded as a life-saving technique for patients suffering from severe respiratory failure, whether acute or chronic, as it facilitates the delivery of oxygen to the lungs by supporting the respiratory process [101]. The development of VAP is influenced by numerous risk factors, including impaired tracheal mucociliary clearance system function, suppression of the cough reflex, the patient’s immune status, advanced age, the presence of comorbidities or trauma and the duration of exposure to the endotracheal tube (ETT) [102]. Indeed, the likelihood of developing ventilator-associated pneumonia (VAP) is increased in patients who have been on mechanical ventilation for a duration exceeding 48 h. Previous studies have demonstrated that patients requiring intubation are subject to a six-to-21-fold increased probability of developing pneumonia [103]. Bacteria stick to the ETT surface and form biofilms, which act as reservoirs for pathogens, increasing the risk of VAP [104].

The management of VAP represents a considerable challenge for healthcare systems, largely due to the high prevalence of multidrug-resistant (MDR) pathogens. These pathogens have been shown to directly increase mortality, treatment failure, and healthcare costs. It has been reported that bacteria causing VAP (e.g., *Pseudomonas aeruginosa, Acinetobacter baumannii* and *Klebsiella pneumoniae*) frequently acquire resistance genes and form biofilms, thereby rendering classical antibiotic treatments ineffective [38,105]. The diagnosis of ventilator-associated pneumonia (VAP) is typically reliant upon culture-based methods and/or molecular techniques, such as filmarray [106]. Nevertheless, the aforementioned approaches are inadequate in providing insights into the process of biofilm formation. Consequently, microscopy-based techniques are frequently utilised, particularly scanning electron microscopy (SEM) and fluorescence in situ hybridisation (FISH), which facilitate the identification and characterisation of biofilms [107]. In addition to the bacteria referenced above, other bacteria frequently isolated from pneumonia cases associated with mechanical ventilation include *Escherichia coli, Staphylococcus aureus, Staphylococcus epidermidis* and *Enterococcus faecium* [108]. Coated endotracheal tubes (ETTs) have been demonstrated to be a highly efficacious strategy in the reduction in bacterial biofilms, which have been identified as a primary factor in the development of VAP. The modification of the surface of the ETT with silver-coated materials has been demonstrated in randomised controlled trials to delay the onset of VAP [109]. Furthermore, the efficacy of the impregnation of antiseptic agents such as chlorhexidine, gentian violet (frequently abbreviated to “gendine” or “gardine”) or triclosan has also been demonstrated. As demonstrated by numerous studies, gendine/gardine-coated tubes have been shown to exhibit a higher level of efficacy in hindering biofilm formation when compared to silver [110]. It has been demonstrated that the utilisation of these tubes can provide a period of up to two weeks of continuous protection against MRSA [111]. The findings of these investigations have demonstrated that there has been a substantial reduction in bacterial colonisation, particularly by pathogenic bacteria such as *Pseudomonas aeruginosa* and *Staphylococcus aureus* [111]. Antimicrobial photodynamic therapy (aPDT) has been identified as a potentially efficacious treatment for ventilator-associated pneumonia (VAP). The process entails the utilisation of photosensitisers in conjunction with light, resulting in the generation of reactive oxygen species (ROS), exemplified by singlet oxygen (_1_O^2^). It has been demonstrated that these ROS have the capacity to oxidise bacterial cellular structures, including lipids, proteins and DNA, and to disrupt the extracellular matrix of biofilms [112,113]. Furthermore, in addition to the elimination of bacterial cells, these ROS have the capacity to disrupt biofilms, particularly those located in the endotracheal tubes (ETTs) of patients receiving ventilatory support. This technique offers a non-invasive, low-resistance alternative for eradicating biofilm structures that are often up to 1000 times more resistant to conventional antibiotics [114]. As has been demonstrated, the photodynamic therapy (PDT) treatment has been found to exert a dual action in the destruction of bacteria within a biofilm. Firstly, it has been shown to exert a direct bactericidal effect and, secondly, it has been found to disrupt the biofilm’s extracellular matrix, thereby facilitating enhanced penetration and resulting in structural degradation [115]. Standard strategies employed to prevent ventilator-associated pneumonia in clinical practice include elevation of the head of the bed, meticulous hand hygiene by healthcare personnel, the administration of thromboembolic prophylaxis, and the implementation of oral care with chlorhexidine (up to a 2% concentration) to effectively decontaminate the oropharyngeal cavity [116,117,118].

### 3.7. Biofilm in Surgical Site Infections

Surgical site infections (SSIs) are defined as postoperative infections that occur subsequent to contamination of the surgical wound or implanted device by endogenous or exogenous bacteria [119]. In less severe cases, the infection remains localised to the dermis; however, in more severe cases, it may involve the subcutaneous layers, internal organs and the circulatory system. The severity of the condition is contingent upon two factors: the surgical intervention undertaken and the characteristics of the wound. In accordance with the established surgical guidelines, an infection is generally categorised as a surgical site infection (SSI) when a bacterial concentration exceeding 100,000 colony-forming units per gram of tissue is identified. This benchmark is widely accepted as a standard measure for distinguishing an infected wound from simple contamination [120]. It has been reported by several studies that SSIs have a considerable impact in hospital settings, in terms of incidence, mortality, prolonged patient hospitalisation, and consequently increased healthcare costs. Furthermore, it is estimated that approximately 80% of these infections are associated with the formation of biofilms, which are often polymicrobial and significantly increase the difficulty of antibiotic treatment [121]. Biofilms are frequently formed by endogenous commensal bacteria such as *Staphylococcus aureus*, *Staphylococcus epidermidis, Enterococcus* spp. and *Escherichia coli*. Exogenous microorganisms have also been identified as a contributing factor to the onset of SSIs, with *Staphylococcus* spp. and *Streptococcus* spp. being the most commonly identified species. These microorganisms can originate from various sources within the healthcare setting, including the operating room environment, air-conditioning systems, surgical instruments, and direct contact with healthcare personnel [121]. According to the criteria established by the Centers for Disease Control and Prevention (CDC), the occurrence of such infections must be within 30 days following surgery or within one year after implantation (e.g., prosthesis, heart valve) [122]. Acute surgical site infections (SSIs) have been demonstrated to occur within 30 days of surgical intervention, and are characterised by a rapid and localised inflammatory response. Such infections are most commonly driven by what is termed ‘planktonic (free-floating) bacteria’ [121,123]. The latter are characterised by their elevated metabolic activity and susceptibility to antibiotics, leading to accelerated tissue destruction and the subsequent formation of purulent exudate (pus) [124]. Chronic surgical site infections (SSIs) are characterised as persistent, difficult-to-treat conditions that are often associated with the formation of biofilms. These biofilms, which may be monomicrobial in their initial phase, characteristically evolve into complex polymicrobial communities over time. The inherent resistance of such polymicrobial biofilms to standard antibiotics has been well-documented, and is a major contributing factor to treatment failure, chronic inflammation, and, consequently, patient mortality [121]. Although the clinical symptoms may resemble those of acute infections, chronic SSIs are generally more severe and involve deeper tissues, particularly in cases associated with implants and prosthetic devices [120]. Evidence-based guidelines advocate a multi-phase approach encompassing preoperative, intraoperative, and postoperative strategies with the objective of preventing surgical site infections (SSIs). The following strategies have been identified as being of paramount importance:

(a) Prior to scheduled surgery, it is vital to diagnose and treat infections located distant from the surgical area (e.g., urinary tract or dermal infections).

(b) It is imperative that the aseptic technique be maintained in all circumstances. Adherence to proper hand hygiene and the use of sterile gowns and gloves are also key components of this protocol.

(c) The prescription of prophylactic antibiotics should be initiated within 60 min (or 120 min for certain agents such as vancomycin) prior to incision in order to ensure adequate tissue concentration [121,125].

### 3.8. Impact of Biofilm Formation on Recurrent Clostridium difficile Infection

*Clostridium difficile* is a Gram-positive, rod-shaped, anaerobic, spore-forming bacterium. It has been identified as the primary etiological agent of diarrhoea in hospital settings following prolonged antibiotic therapy and in the presence of patient-related risk factors, such as immunosuppression and other comorbidities [126]. It is estimated that approximately half a million patients acquire *C. difficile* infection (CDI) in US hospitals each year, with an associated mortality rate of 5–6% [127]. The pathogenesis of *C. difficile* begins when the highly resistant endospores are ingested and survive the acidity of the stomach. These endospores then germinate into vegetative cells within the intestinal tract, leading to bacterial colonisation. The secretion of toxins A and B by vegetative cells has been identified as the primary cause of damage to the intestinal epithelial barrier, resulting in an inflammatory response that can manifest as diarrhoea or fulminant pseudomembranous colitis [128]. CDI is a particular concern due to its propensity for recurrence, which occurs in 20% to 25% of patients within 4 weeks of completing anti-*C. difficile* treatment. Furthermore, recurrent patients have a higher risk of death compared to those without recurrence [129]. Adhesion of *C. difficile* to the gastrointestinal mucosa represents a pivotal preliminary phase in the process of colonisation, frequently resulting in the development of biofilms, which act as a protective reservoir for the bacteria. This process, in concert with subsequent biofilm maturation, has been well documented in in vitro experiments using confocal laser scanning microscopy and scanning electron microscopy [130].

In 2019, the Centers for Disease Control and Prevention classified *C. difficile* as an “urgent threat” pathogen, owing to its status as a primary cause of healthcare-associated infections within the United States [131].

Antibiotic resistance in *C. difficile* is closely associated with its ability to form biofilms, although it should be noted that not all strains exhibit this capacity. The formation of biofilms is strain-dependent and influenced by environmental conditions within the intestinal microbiota, as well as by regulatory pathways involving the second messenger cyclic di-guanosine monophosphate (c-di-GMP). This molecule plays a central role in the transition from the motile to the sessile state, promoting biofilm formation through the downregulation of genes encoding flagellar components [132].

The development of biofilms is favoured by a number of factors, including, but not limited to, prolonged antibiotic exposure, dysbiosis, and environmental stresses such as limited nutrients and altered pH levels. In vitro studies, encompassing horizontal growth in microtiter plates, vertical growth on solid surfaces, as well as growth in bioreactors and on solid media, have demonstrated that *C. difficile* biofilms manifest as thin and viscous structures wherein bacterial cells are able to differentiate into persister cells [133]. It is noteworthy that, in contrast to numerous other bacterial species, *C. difficile* biofilms do not manifest the distinctive mushroom-like architecture characteristic of most bacterial biofilms. In its planktonic form, *C. difficile* exists as free, motile cells equipped with flagella and is more susceptible to host immune defences and antibiotic treatment. In contrast, bacteria that form biofilms establish structured communities which are characterised by: slower growth, greater tolerance to antimicrobial agents and enhanced resistance to host immune responses. This contributes to the persistence and recurrent infection of the organism [134,135].

The main factors that allow *C. difficile* to colonise, adhere to, and damage the intestinal epithelium are: toxin A (TcdA), toxin B (TcdB), actin-ADP-ribosylating toxin (CDT), and surface adhesion proteins. Toxin A is a large enterotoxin, typically comprising 2710 amino acids, that causes fluid accumulation in the ileum and damage to the intestinal mucosa. Toxin B is a potent cytotoxin consisting of 2366 amino acids, which is typically 100 to 1000 times more potent than Toxin A in damaging cultured cells [136]. *C. difficile* employs accessory gene regulator (Agr) systems, specifically Agr1 and Agr2, to regulate the production of toxins and virulence. Distinct roles have been identified for each of these systems. Both toxins bind to intestinal cells, cleave, and translocate into the cytosol. They inactivate Rho- and Ras-GTPases via mono-O-glucosylation, disrupting tight junctions, inducing apoptosis and phagocytosis, and causing diarrhoea and colitis [137]. The standard methods for diagnosing *C. difficile* infection (CDI) rely on the presence of diarrhoea (unformed stool) in conjunction with laboratory confirmation of *C. difficile* toxins in stool samples. Prolonged use of antibiotics has been demonstrated to cause an imbalance in the intestinal microbiota, promoting the colonisation and subsequent infection by *C. difficile*, which has been observed to exhibit increasing mechanisms of antibiotic resistance to clindamycin, fluoroquinolones, and metronidazole. Antibiotic treatment failure occurs in about 30% of patients, and antibiotics can change the gut microbiome, leading to relapse in approximately 25% of patients after treatment [132,138].

## 4. Biofilm as a Driver of Antibiotic Resistance

Biofilms have been demonstrated to be functional structures that facilitate the protection of microbial communities against the immune defences of the host organism. Biofilms rely on quorum-sensing signals for communication during all stages of development, including adhesion, maturation and dispersion. These signals coordinate bacterial activity and trigger immune responses. In addition, they have been shown to increase the resistance of such communities to antibiotic treatments [52]. The EPS matrix creates a protective microenvironment that significantly decreases bacteria’s susceptibility to antimicrobial agents compared to planktonic cells [5]. One of the principal mechanisms that contributes to antibiotic resistance in biofilms is the limited penetration of antibiotics through the EPS matrix. The matrix functions as a physical and chemical barrier, impeding the diffusion of antimicrobial compounds and sequestering antibiotic molecules. This may result in the inactivation of the antibiotic molecules before they can reach the deeper layers of the biofilm [139]. Consequently, cells located in the inner regions of the biofilms may be exposed to sub-inhibitory concentrations of antibiotics, thereby enabling their survival and promoting the selection of antibiotic-resistant phenotypes [14]. Furthermore, cells located in the deeper layers of the biofilm frequently demonstrate diminished metabolic activity or reduced growth rates, a consequence of restricted nutrient availability. It is noteworthy that a significant proportion of antibiotics primarily target actively dividing bacteria. Consequently, these metabolically inactive or slow-growing cells exhibit increased tolerance to antimicrobial treatments [52]. Another significant factor that must be given full consideration is the presence of persister cells, a subpopulation of phenotypic variants capable of surviving antibiotic exposure without possessing genetic resistance. It has been observed that these cells can persist in a state of dormancy during antibiotic treatment, and upon the removal of antimicrobial pressure, they undergo a process of repopulation within the biofilm [140]. This phenomenon has been demonstrated to be a contributing factor to both infection relapse and chronicity. As demonstrated in the preceding studies, these cells have been identified in a variety of bacterial and fungal species, including *P. aeruginosa, E. coli, S. aureus, C. albicans, A. baumannii* and *B. cereus* [141]. Furthermore, the structural organisation of biofilms—characterised by elevated levels of cellular density and close spatial proximity among microorganisms—has been demonstrated to augment the frequency of point mutations and the mechanisms of horizontal gene transfer via conjugation, transformation, and transduction [142]. In addition to these well-established mechanisms, a fourth gene transfer mechanism, mediated by extracellular vesicles produced by both Gram-positive and Gram-negative bacteria, has been proposed. These vesicles transport various bacterial cargo, e.g., transmembrane proteins, enzymes, toxins, peptidoglycan, lipoproteins, lipoteichoic acids, and nucleic acids, and can be internalised by other bacterial cells through membrane fusion. For instance, the transfer of plasmids between phylogenetically distant bacteria has been demonstrated to occur via vesicles [143]. These plasmids have been demonstrated to confer resistance to β-lactam antibiotics, and the bacteria involved include *Escherichia coli, Salmonella enterica, Pseudomonas aeruginosa* and *Burkholderia cepacia* [144]. Furthermore, it has been demonstrated that bacterial cells released from biofilms retain their antibiotic-resistant properties, indicating that this resistance persists even after the cells transition to a planktonic state [145]. As previously highlighted, quorum sensing (QS) is recognised as a key cell–cell communication mechanism in biofilms. The coordination of bacterial behaviour is facilitated by signalling molecules, including acyl-homoserine lactones (AHLs) in Gram-negative bacteria and oligopeptides in Gram-positive bacteria [42]. In addition, QS has been demonstrated to play a regulatory role in the activation of efflux pumps, including MexAB-OprM, MexEF-OprN, and MexCD-OprJ. These pumps have been demonstrated to contribute to the development of antibiotic resistance through the enhanced extrusion of antimicrobial agents [146]. Whilst it is now widely accepted that biofilms play a role in the development of antibiotic resistance, further research is required to establish the correlation between a strain’s inherent genetic resistance and its capacity to form a biofilm. A recent study showed an unexpected relationship between antibiotic resistance in *S. maltophilia* and biofilm formation. The study found that more multidrug-resistant clinical isolates had less biofilm, and more susceptible isolates had more biofilm [147]. These findings suggest that biofilm formation might be a way for susceptible strains to survive, not just a result of increased resistance.

## 5. Biofilm Prevention and Control Strategies

Antibiotic resistance is widely regarded as one of the most critical challenges of the 21st century. In recent decades, considerable investment has been directed towards research efforts aimed at combatting infections associated with biofilms. A particular emphasis has been placed on the prevention of biofilm formation to reduce the incidence of infections caused by multidrug-resistant bacteria, which are often associated with high mortality rates (Table 1). These bacteria, including *P. aeruginosa*, *A. baumannii*, *E. coli* and *S. aureus*, are often designated as “superbugs” due to their ability to disseminate globally, exhibiting high-risk clones that combine extreme drug resistance with a substantial capacity to form biofilms, thereby rendering the infections challenging to treat.

A fundamental strategy for hindering the formation of biofilms involves the implementation of engineered anti-adhesive surfaces [148]. Examples of such material include zwitterionic substances, silver-coated surfaces, silver oxynitrate and copolymers, which find widespread use in medical device coating and bacterial attachment inhibition [149]. In addition, metallic nanoparticle (NP) formulations have been engineered to impede bacterial adhesion, a property deemed to be critical in preventing the formation of biofilms. These nanoparticles are frequently amalgamated with enzymes, including α-amylase and glycoside hydrolases, which are capable of degrading 1,4-glycosidic bonds within the biofilm matrix [150]. Despite the absence of intrinsic antimicrobial properties, nanoparticles have been extensively explored as delivery systems, with the aim of enhancing the penetration of antibiotics within biofilms. Among these elements, gold (Au), silver (Ag), and zinc (Zn) nanoparticles have received the most attention in research focused on anti-biofilm applications [151].

**Figure 3:**
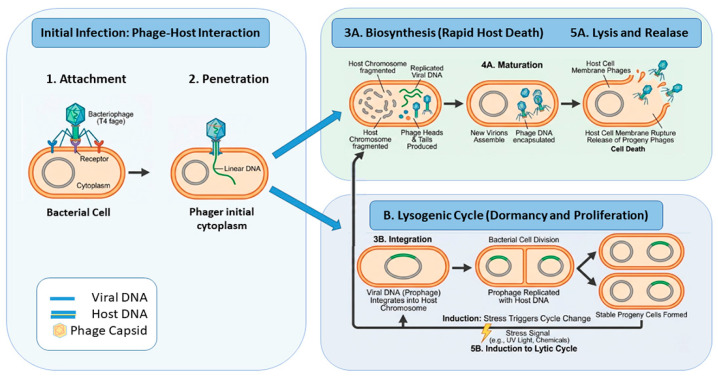
Comparison between the lytic and lysogenic cycles of bacteriophages. The lytic cycle is characterised by rapid viral replication, host cell lysis, and release of progeny virions. In contrast, the lysogenic cycle involves integration of the phage genome into the host chromosome, allowing vertical transmission until induction triggers the lytic pathway.

It is important to note that a number of other promising strategies have been identified, including quorum-sensing (QS) inhibitors, which are also known as quorum-quenching (QQ) agents. The efficacy of these compounds in impeding bacterial communication has been demonstrated, thereby inhibiting biofilm formation and enhancing antibiotic efficacy [152]. A number of naturally derived compounds have been shown to exert anti-QS properties, including cinnamaldehyde (a constituent of cinnamon), flavonoid derivatives (e.g., baicalein and quercetin), and eugenol. Furthermore, the role of enzymes in relation to biofilms has been identified as a critical factor in the management and control of such structures [153]. Furthermore, the role of enzymes in relation to biofilms has been identified as a critical factor in the management and control of such structures. For instance, nucleases such as deoxyribonuclease I (DNase I) have been demonstrated to possess the capacity to degrade extracellular DNA within the biofilm matrix. This process has been shown to result in a reduction in the structural integrity of the biofilm and an increase in its permeability to antimicrobial agents [154]. A further promising strategy entails the utilisation of bacteriophages, which possess the capacity to selectively infect and kill bacteria and are thus being investigated for their potential in disrupting biofilms. Lytic phages have the capacity to produce enzymes that are capable of degrading the exopolysaccharide components of the EPS matrix and the bacterial capsule, thereby weakening the structural integrity of the biofilm (Figure 3) [155].

Furthermore, a significant number of other phage-derived enzymes, including hydrolases such as glucanase, levosidase, sialidase, rhamnosidase, xylosidase and hyaluronidase, have been demonstrated to possess the capacity to compromise the structural integrity of biofilms. This process enables the infiltration of phages with the enclosed bacterial cells and subsequently enhances the immune system’s ability to destroy bacterial cells. Antimicrobial peptides (AMPs) represent a class of naturally occurring molecules that demonstrate broad-spectrum activity against Gram-positive and Gram-negative bacteria, as well as fungi. A substantial number of AMPs act during the early stages of biofilm formation by inhibiting bacterial adhesion through the downregulation of genes involved in attachment [52]. It has been demonstrated that other antimicrobial peptides (AMPs), including bacteriocins, are able to compromise bacterial cell membranes. Furthermore, it has been demonstrated that antimicrobial peptides (AMPs) possess the capacity to increase membrane permeability, thereby enhancing the efficacy of antibiotics and disrupting quorum sensing. Furthermore, it has been demonstrated that AMPs are capable of contributing to the degradation of the exopolysaccharide matrix [156]. The application of electric current to disrupt the integrity of the biofilm matrix with the aim of detaching bacterial cells from surfaces has been identified as a promising strategy for treating biofilm-related infections. Moreover, the combination of low-intensity electric fields with antibiotics has been demonstrated to result in a substantial increase in the destruction of biofilm-associated bacteria, often rendering them susceptible to low concentrations of antimicrobials that are effective against free-floating (planktonic) bacteria [157].

## 6. Discussion and Conclusions

Microorganisms are able to aggregate and organise into structured communities embedded within an exopolysaccharide-based matrix, which shields them from antibiotic activity and host immune defences, thereby sustaining infections that are notoriously difficult to eradicate. Moreover, bacteria within a biofilm community interact and communicate in ways that free-living bacteria cannot, creating more complex ecosystems. The present review examines the physicochemical properties and the formation of biofilms by clinically significant pathogens, highlighting their clinical relevance and associated risks, particularly in nosocomial settings. Biofilm-related infections impose significant financial burdens on healthcare systems and can lead to patient morbidity and mortality. Research is focusing on finding new ways to combat infections caused by such pathogens. In particular, the following three aspects of biofilm research are of particular interest: firstly, the prevalence of such infections, secondly, the aetiology of the condition, and thirdly, new approaches to the treatment thereof. In the context of combating infections caused by biofilm-forming pathogens, it is essential to acknowledge the capacity of biofilms to adhere to both abiotic and biotic surfaces. These biofilms frequently give rise to the formation of polymicrobial communities, which can result in chronic infections that are difficult to treat due to multiple mechanisms of antibiotic resistance. Furthermore, bacterial cells within biofilms communicate through QS, a process that drives the development of the extracellular matrix and enables coordinated, high-density intercellular communication. This organisation facilitates the exchange of genetic material, thereby promoting the acquisition and dissemination of antibiotic resistance genes. Secondly, biofilms express efflux pump systems that actively expel antimicrobial agents, thereby reducing their susceptibility and rendering the biofilm structure highly recalcitrant to treatment. Of further significance is the observation that persister cells within the biofilm do not exhibit genetic resistance but rather phenotypic tolerance, entering a dormant state that protects them from the effects of antibiotics. In response to these multifarious challenges, there is an urgent need to develop effective prevention and treatment strategies that target biofilm-associated infections. Despite the encouraging advancements witnessed over the past two decades, including the development of anti-biofilm surfaces, the utilisation of nanoparticles as antibiotic delivery systems, quorum-sensing inhibitors, and enzymatic and antimicrobial peptide-based approaches capable of reducing biofilm biomass and enhancing antibiotic efficacy, further research is necessary to fully explore the potential of these methodologies. For instance, QSIs represent a promising approach for targeting bacteria’s communication pathways to modify biofilm formation. At the same time, other agents, such as bacteriophage, are being studied as a way to break down biofilm and make antibiotics more effective. Notwithstanding the advances in therapeutic options, many of these new treatments encounter substantial barriers to utilisation in clinical practice, including a paucity of clinical validation and safety concerns. This underscores the necessity for further research to develop more effective and clinically applicable strategies.

## Figures and Tables

**Table 1:** Strategies for preventing biofilm formation and their mechanisms of action **^#^**.

Anti-Biofilm Strategy	Mechanism of Action	Pathogens	Infections	References
Nanoparticles	Surfaces exhibit anti-adhesive properties either intrinsically or through antibiotic coatings. Metallic nanoparticles (NPs), including silver (Ag), gold (Au), zinc (Zn), and other agents (e.g., gendine), delay microbial colonisation and enhance antibiotic efficacy.	MRSA, *P. aeruginosa*	Ventilator-associated pneumonia, infections related to medical devices, and untreated prostheses.	[16,118,121,125]
Enzymes	Degradation of glycosidic bonds in the polysaccharide matrix by enzymes and extracellular DNA (eDNA) by DNase I. reduces the structural integrity of the biofilm.	*S. aureus*, *P. aeruginosa*	Device-associated infections and pulmonary infections in cystic fibrosis patients.	[125]
Antimicrobial Peptides (AMPs)	Inhibit bacterial adhesion by reducing adhesion genes, disrupting cell membranes and degrading the EPS matrix.	Gram-positive bacteria, Gram-negative bacteria, and fungi (*Candida* spp.)	Chronic persistent infections and polymicrobial biofilm-associated infections.	[16]
Quorum-Sensing Inhibitors	Interfere with bacterial communication systems to inhibit both biofilm formation and toxin production.	*C. difficile*, *P. aeruginosa*, *S. aureus*.	Gastrointestinal infections (CDI) and recurrent nosocomial infections.	[63]
Bacteriophages	Engineered bacteriophages selectively infect and lyse bacterial populations, resulting in the disruption of biofilms.	Specific phage-targeted pathogens (high selectivity).	Multidrug-resistant biofilm infections that are difficult to eradicate.	[16,125]
Physical Methods	The application of an electric current promotes the detachment of biofilms, while photodynamic therapy (aPDT) generates reactive oxygen species (ROS), which oxidise cellular structures.	MDR pathogens (e.g., *A. baumannii, K. pneumoniae*)	Infections associated with endotracheal tubes (VAP) and contaminated abiotic surfaces.	[16]

**^#^** Anti-biofilm strategies encompass a wide range of approaches, including the application of nanoparticles to medical surfaces, the development of next-generation materials with intrinsic antimicrobial activity, the use of enzymes for cellular degradation, and viral and physical approaches. The efficacy of these strategies is demonstrated in the treatment of a wide range of bacteria and fungi responsible for major infections.

## Data Availability

Data are available on request.
